# Bilateral ovarian micrometastatic adenocarcinoma upon prophylactic oophorectomy concurrent with low anterior resection for rectal cancer

**DOI:** 10.1186/s12957-017-1115-6

**Published:** 2017-02-07

**Authors:** Robin Irons, Erin McIntosh, Alexandre Hageboutros, David Warshal, Steven McClane

**Affiliations:** Department of Surgery, Cooper University Hospital and Cooper Medical School of Rowan University, Three Cooper Plaza, Suite 403, Camden, NJ 08103 USA

**Keywords:** Oophorectomy, Colorectal cancer, Prophylaxis, Metastasis, Ovarian cancer

## Abstract

**Background:**

This case report draws attention to the debated role of prophylactic oophorectomy in women undergoing definitive surgical resection of colon and rectal cancers. It can be challenging to discern the indications and appropriate patient population for this procedure based on the current literature. Potential benefits include treatment and prevention of metastatic disease, preventing development of primary ovarian cancer, and prolonging survival. Negative effects include an increase in operative time and potential morbidity, development of osteoporosis, the risk of cardiac events, and decreasing sexual function. Multiple patient factors such as age, menopausal status, patient preference, presence of hereditary conditions, exposure to radiation, site, and stage of disease should be considered.

**Case presentation:**

We present a case in which a premenopausal 49-year-old female underwent a prophylactic bilateral salpingo-oophorectomy concurrently with a low anterior resection following neoadjuvant chemoradiation for clinical stage III rectal cancer. On pathologic examination, resection margins and all 14 lymph nodes harvested were negative for malignancy. Interestingly, she was found to have micrometastatic adenocarcinoma in the bilateral ovaries which had appeared grossly normal at the time of surgery.

**Conclusions:**

After consideration of the current literature, patient preference, and our clinical judgment, our patient ultimately had a therapeutic effect after undergoing prophylactic bilateral oophorectomy concurrently with a low anterior resection for rectal cancer. The addition of prophylactic oophorectomy in a select population, specifically women 50 years of age or younger and/or women who are in the premenopausal state, may carry a survival benefit in the setting of definitive surgical resection of colon and rectal cancers.

## Background

In this case report, we present a patient who underwent a prophylactic oophorectomy (PO) during a low anterior resection for clinical stage III rectal cancer after neoadjuvant chemotherapy and radiation who was found to have micrometastatic adenocarcinoma in the bilateral ovaries. The role of PO in primary colorectal cancers is controversial. Proposed benefits include decreasing disease recurrence, preventing development of primary ovarian cancer, and prolonging survival; however, there is limited data to support these proposals [1–3]. While the risk of immediate complications from the procedure are minimal, patients may experience long-term negative consequences including decreased sexual function, development of osteoporosis, and increased risk of cardiac events [4]. These negative consequences are largely offset by the use of exogenous estrogen therapy. The balance of the risks and benefits are affected by multiple patient factors including patient preference, age, menopausal status, presence of hereditary conditions, site, and stage of disease.

## Case presentation

Our patient is a 49-year-old, previously healthy, premenopausal female who presented to an outside institution’s emergency department with a chief complaint of new onset, left lower quadrant pain and several weeks of constipation. She underwent her first colonoscopic evaluation which demonstrated a near obstructing mass at the rectosigmoid junction. She was transferred to our institution for comprehensive cancer care.

**Fig. 1:**
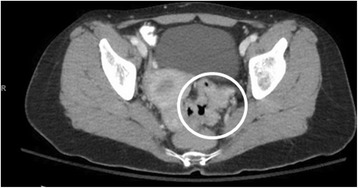
Preoperative CT scan demonstrating a focal area of irregular wall thickening and associated luminal narrowing at the level of the rectosigmoid junction

**Fig. 2:**
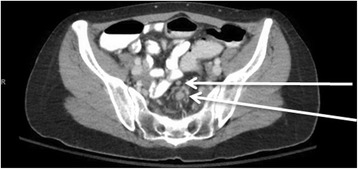
Preoperative CT scan demonstrating multiple, borderline, perirectal lymph nodes with the largest measuring 1.0 cm

On admission interview, she reported that her obstructive symptoms had been progressively escalating, with her last bowel movement over a week ago. She denied personal history of cancer. Her family history was significant for a grandmother with gastric cancer in her late 60s. Pathology identified the mass as a moderately differentiated, invasive adenocarcinoma. She underwent a computed tomography (CT) scan of the chest, abdomen, and pelvis for staging. Imaging revealed wall thickening at the rectosigmoid junction and luminal narrowing (Fig. 1). Suspicious perirectal lymph nodes were present, but there was no evidence of distant metastasis (Fig. 2). Due to her progressive obstructive symptoms and abdominal pain, she was taken to the operating room for a diverting loop ileostomy and a rigid sigmoidoscopy which demonstrated the distal border of the lesion at approximately 9 cm from the anal verge.

**Fig. 3:**
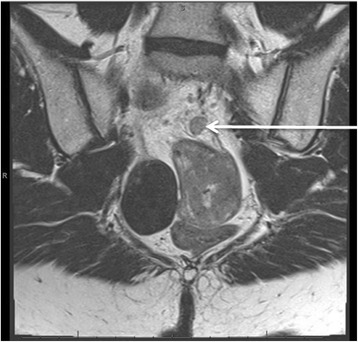
Preoperative MRI demonstrating the largest (0.9 cm) of multiple perirectal lymph nodes

During her initial admission, she was evaluated by our hematology/oncology and our radiation oncology department for outpatient neoadjuvant therapy. She underwent an MRI of the pelvis to further assist in staging which displayed a 4.6 cm mass located in the upper rectum that extended into the muscularis propria, classifying it as a T3b lesion. Enlarged superior rectal lymph nodes suspicious for malignancy were also noted (Fig. 3). Given her clinical diagnosis of stage III adenocarcinoma of the rectum, she was deemed a candidate for chemotherapy with 5-fluorouracil continuous infusion and concurrent radiation followed by definitive surgical resection.

**Fig. 4:**
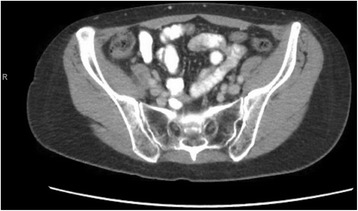
Post neoadjuvant therapy CT scan demonstrating resolution of perirectal lymph node enlargement

Ten weeks after completion of preoperative chemoradiation, she underwent a repeat CT scan (Fig. 4). She had an appropriate response as evident by decreased rectal wall thickening and resolution of perirectal lymph node enlargement to the extent that nodes were no longer visualized.

She then underwent a laparoscopic low anterior resection. Our gynecology team was consulted prior to the procedure for a hysterectomy, as the patient was known to have a 4 cm posterior leiomyoma. Given her age, family history, recent chemotherapy, and pelvic radiation, the gynecologist felt she would benefit from a bilateral salpingo-oophorectomy which she had also consented for. On gross inspection, both ovaries appeared normal.

**Fig. 5:**
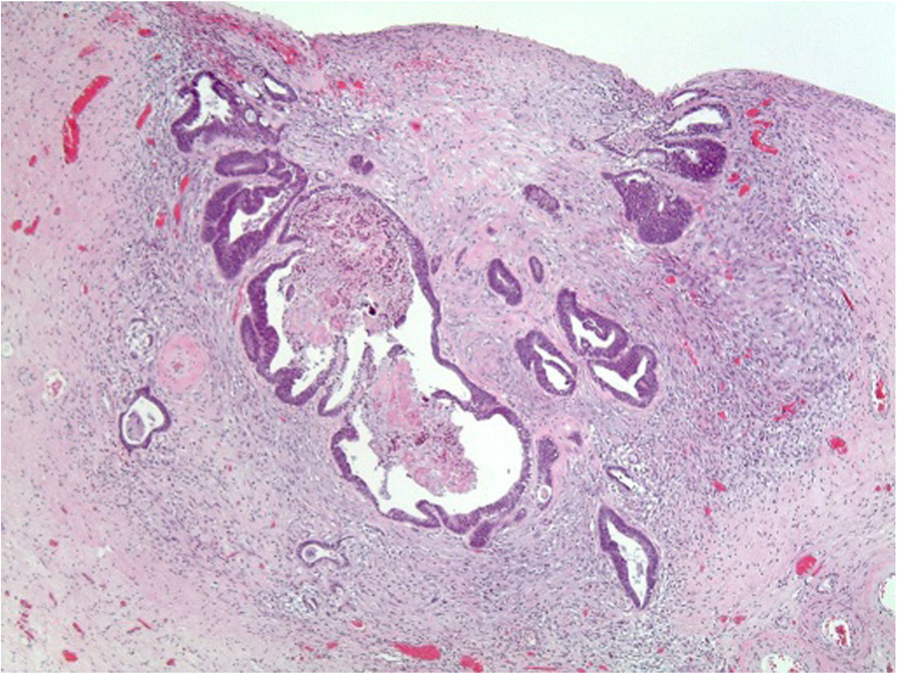
×50 H&E stain. A small (up to 2.5 mm) focus of adenocarcinoma is present in the cortex near the surface of the right ovary

Pathology revealed moderately differentiated, ulcerated adenocarcinoma of the rectum with a depth of invasion to 0.1 mm from the peritoneal surface (Fig. 5). Proximal, distal, and radial resection margins were clear of malignancy and 0 of the 14 nodes harvested contained malignancy. Pericolorectal adipose contained tumor deposits and there was focal extramural vascular invasion. A 3.7 cm subserosal leiomyoma was present on the posterior aspect of the uterus which was consistent with gross examination. Of greater interest was the presence of metastatic rectal adenocarcinoma in the cortex of the left and right ovaries. Immunohistochemical testing of the rectal tumor for mismatch repair genes, *MSH2*, *MLH1*, *MSH6*, and *PMS2*, associated with Lynch syndrome, was not suggestive of the disease.

## Discussion

In women undergoing colorectal cancer resection, concurrent PO is not generally recommended or performed, yet it does need to be considered. These women carry a risk of developing metastatic disease to the ovaries in addition to their baseline risk of developing primary ovarian cancer. PO concurrently with definitive bowel resection has been proposed, as the ovaries are easily accessible via laparoscopy or laparotomy and resection carries minimal surgical risks. Current literature provides some insight into the risks women face with ovarian preservation, but guidelines for PO in this population have not been established.

### Metastasis disease to the ovaries

The rate of metastasis of colorectal cancer to the ovaries in an average woman ranges from 1.4 to 4% [1, 5]. This rate significantly increases when evaluating women prior to menopause or at age 40 years or less [1, 6, 7]. Mackeigan et al. reviewed the clinical course of 484 women with colorectal cancer seen at Ferguson-Droste-Ferguson Hospital over an 8-year period. One hundred thirty-seven underwent oophorectomy with 133 prophylactically performed for grossly normal or suspicious involvement [7]. Eight (6%) of the women were found to have metastatic disease to the ovaries. Premenopausal status was found to be associated with a higher rate of metastatic disease to the ovaries with 22% of premenopausal women affected.

Walton et al. reviewed 37 women with colorectal cancer who were less than 40 years old. Metastasis to the ovaries was noted in 5 (13.2%) of these women. Recalde et al. identified 18 females diagnosed with colorectal cancer at age 35 or younger. Four (22%) developed metastatic disease to the ovaries [5]. Blamey identified 882 women who underwent resection of a primary colorectal cancer. Thirteen (1.4%) later required oophorectomy for ovarian recurrence [1]. The mean age of those who developed metastases to the ovaries was 51 years compared to 59 years in those who did not, which met a statistical significance. The lower incidence of ovarian recurrence in this study may be related to imperfections in long-term patient follow-up; therefore, the true incidence is not known.

### Incidence of primary ovarian cancer

According to the 2010–2012 Surveillance, Epidemiology, and End Results Program, the average woman in the USA has a 1.3% chance of developing a primary ovarian cancer in their lifetime with a 5-year survival rate of 46.2%. Age is a significant risk factor for the development of primary ovarian cancer with the median age of diagnosis being 63 years and 88% being diagnosed after the age of 45 [8]. Other contributing factors include but are not limited to obesity, reproductive history, use of hormone replacement therapy, and environmental factors [9].

McCredie et al. sought to establish the risk of developing a new primary cancer among those with a history of colorectal cancer using 20 years of data from the New South Wales Central Cancer Registry [3]. Patients with a diagnosis of invasive cancer of the colon or rectum who had survived beyond 2 months were identified. Patients who were diagnosed with a second primary within the first 2 months were omitted as they were thought to have two synchronous cancers. Compared to the general female population, women with colon cancer were found to be 2.8 times more likely to develop a primary ovarian cancer while those with rectal cancer were only 1.1 times more likely. Hereditary predispositions, hormonal factors, and dietary factors may contribute to this increased risk [3].

While high-dose radiation is known to increase the risk of developing ovarian cancer, the effect of the lower doses of radiation, such as those used in neoadjuvant therapy for colorectal cancer, is unclear. Recently, SEER data was utilized to retrospectively review the incidence of ovarian cancer over a 10-year period in 46,404 patients with rectal or rectosigmoid cancer [10]. Twenty (0.15%) of the 13,099 patients who were treated with beam radiation were diagnosed with ovarian cancer compared to 91 (0.27%) of the 33,305 patients who did not receive radiation therapy. Authors found a 44% decreased risk of ovarian cancer in the group who received radiation compared to the non-irradiated group. Pitfalls of this study include its retrospective design, use of correlation among variables, lack of information on patients’ exposure to chemotherapy, radon, and total background exposures.

Based on these studies, risk factors for the development of primary ovarian cancer appear to include a history of colon cancer. Rectal cancer has not been demonstrated to increase risk of primary ovarian cancer which may be secondary to a protective effect that might occur with low-dose radiation.

### Ovarian cancer in grossly normal ovaries

While gross abnormalities of the ovary can be appreciated upon visual inspection and palpation, not all metastatic ovarian disease are grossly apparent. Burt reviewed 493 cases of colorectal cancer treated at Presbyterian Hospital in New York over a 14-year period and found 17 cases of metastatic adenocarcinoma to the ovaries [11]. Four (24%) had grossly normal ovaries on intraoperative inspection. In the previously mentioned Mackeigan study, 50% of women found to have ovarian metastases at the time of surgery had grossly normal ovaries [7].

### Impact on survival

Impact of PO on survival within this population has not been clearly established due to limited sample sizes. Survival benefits may be affected by multiple patient characteristics including age, presence of hereditary syndromes, and stage of disease. The Mayo Clinic conducted a prospective, randomized trial encompassing 149 patients with Dukes’ stages B or C colorectal cancer [12]. Patients were randomized to either PO or no oophorectomy. Survival curves suggested a potential survival benefit between 2 and 3 years post intervention, but this was not found to be a statistically significant difference and it did not persist at 5 years post intervention.

A retrospective review at the Mayo Clinic did not show a statistically significant survival advantage for 75 women who underwent oophorectomy when compared to the 496 patients in the control group [13]. Of interest, when patients were placed in age-based subgroups, the 5 women who were less than 50 years of age and had undergone PO survived more than 5 years. Cutait et al. examined a group of 335 women who underwent surgical resection of their colorectal cancer [2]. Two hundred one patients were selected based on individual surgeon’s preference and judgment to undergo PO at the time of their initial definitive bowel resection. Four patients were found to have ovarian metastasis at the time of surgery. Disease-free survival, overall survival, and recurrence were evaluated with greater than 5-year follow-up achieved in 93% of the study population. There was no difference found in survival or recurrence when patients were stratified by menopausal status.

## Conclusions

Determining the role of PO among women with resectable colorectal cancer remains difficult due to the limited amount of statistically significant data and the multiple patient variables at play. While older women are at a higher risk of developing a primary ovarian cancer, Walton, Blamey, and Recalde showed that women of a younger age were at a higher risk of having metastatic ovarian disease [6]. Additionally, Mackeigan showed an association with premenopausal status [7]. Although an improvement in survival is suggested in the women under 50 years of age, determining the effects of PO on survival requires a larger collection of data.

Menopausal status, age, chemotherapy, and radiation exposure, reproductive goals along with perspective on hormone replacement therapy should also be taken into consideration when counseling a patient. While the oophorectomized state is associated with increased risk of osteoporosis, coronary artery disease, decreased cognitive, and sexual functions, hormone therapy may help lessen these risks [4].

Given the not insignificant occurrence of incidentally found metastatic colorectal cancer to the ovaries in women undergoing resection, in addition to the risk of developing a primary ovarian cancer in years following resection, should prophylactic oophorectomy be offered to all women at the time of colorectal surgery? But more relevant may be this question in younger women, either under 50 years of age or in the premenopausal state, particularly those with colon cancer or with rectal cancer not receiving radiation therapy. These women, as well as those with strong family histories of other cancers, should certainly be strongly encouraged to consider undergoing concomitant oophorectomy.

Our patient was less than 50 years of age at presentation and had met her reproductive goals. She was premenopausal and underwent chemotherapy and pelvic radiation as part of her adjuvant therapy which often results in loss of ovarian function. The purpose of her surgical resection was to eliminate the risks of developing a primary or metastatic ovarian cancer, understanding the immediate and long-term risks of the oophorectomized state. Fortunately for her, the removal of her grossly normal appearing ovaries eliminated the only metastatic source of disease recurrence and should by all means improve her survival.

## Abbreviations

CT: Computed tomography
PO: Prophylactic oophorectomy
